# Evidence for C1q-mediated crosslinking of CD33/LAIR-1 inhibitory immunoreceptors and biological control of CD33/LAIR-1 expression

**DOI:** 10.1038/s41598-017-00290-w

**Published:** 2017-03-21

**Authors:** Myoungsun Son, Betty Diamond, Bruce T. Volpe, Cynthia B. Aranow, Meggan C. Mackay, Frances Santiago-Schwarz

**Affiliations:** 1 Center for Autoimmune and Musculoskeletal Diseases, The Feinstein Institute for Medical Research, Northwell Health, Manhasset, NY 11030 USA; 2 Center for Biomedical Science, The Feinstein Institute for Medical Research, Northwell Health, Manhasset, NY 11030 USA

## Abstract

C1q collagen-like region (CLR) engaging and activating the LAIR-1 inhibitory immunoreceptor represents a non-complement mechanism for maintaining immune quiescence. Given the binding promiscuity of C1q’s globular region (gC1q), we hypothesized that C1q concurrently associates with distinct inhibitory immunoreceptors to produce C1q-mediated modulatory networking. Like LAIR-1, CD33 inhibitory immunoreceptors are highly expressed on monocytes. Binding CD33 restricts cell activation/differentiation; however, natural ligands for CD33 remain elusive. CD33 has IgC2-like domains potentially recognized by gC1q. Thus, we asked whether C1q binds to CD33 and if C1q mediates CD33/LAIR-1 crosslinking. Our findings demonstrate that C1q and gC1q interact with CD33 to activate its inhibitory motifs, while CLR does not. Whole C1q is required to crosslink CD33 and LAIR-1 and concurrently activate CD33/LAIR-1 inhibitory motifs. While C1q binds CD33C2 domains, decreased C1q-CD33 interactions resulting from sialic acid masking of CD33C2 domains suggests a process for regulating C1q-CD33 activity. Consistent with defective self-tolerance, CD33/LAIR-1 expression is reduced in systemic lupus erythematosus (SLE) myelomonocytes. The anti-inflammatory cytokine M-CSF, but not DC growth factors, sustains CD33/LAIR-1 expression on both healthy and SLE cells suggesting further biological control of C1q-CD33/LAIR-1 processes.

## Introduction

Apart from C1q binding to the Fc (constant) region of antigen (Ag) complexed IgG or IgM to activate the classical complement pathway, C1q controls innate and adaptive immunity^1^. C1q facilitates phagocytosis and regulates immune cell differentiation, survival, migration, and cytokine secretion^1–6^. The full length molecule is assembled from 18 polypeptide chains (6A, 6B and 6C chains). Each chain contains a C-terminal globular module (gC1q) and an N-terminal collagen-like Gly-Pro-Hyp (GPO) repeat region (CLR). Assembly of the A, B, C, chains into heterotrimer subunits is followed by organization into a 460 kDa hexamer displaying a distinct globular head region and a collagen tail region. C1q is abundant in serum and is amongst the most positively charged of serum proteins. Based on the ability of C1q (especially its globular region) to interact with multiple partners, it is classified as a pattern recognition molecule^1^.

In addition to C1q’s role in clearing immune complexes and apoptotic bodies for preventing unwarranted inflammation and autoimmunity, an important evolving idea is that C1q-C1q receptor inhibitory action on immune cells may directly induce tolerance and inhibit autoimmunity^7–10^. In support of this premise, C1q suppresses the ability of freshly isolated peripheral blood (PB) monocytes to differentiate into dendritic cells (DCs) and inhibits IL-12 production by monocytes^8, 11, 12^. However, understanding of membrane-proximal or intracellular signaling processes involved in such C1q-mediated inhibitory signaling is limited. A growing number of cell-associated receptors for both gC1q and CLR, including CD93 (C1qRp), CD35 (CR1), gC1qR (p33), α2β1 integrin, calreticulin (cC1qR), CD91, SCARF-1 and RAGE, have been associated with C1q’s complement independent functions, particularly with its ability to facilitate uptake of extracellular material into phagocytic cells^1, 6, 10, 13^. Because these C1q receptors do not exhibit intracellular inhibitory signaling domains^11^ and lack direct inhibitory action like receptors bearing immunoreceptor tyrosine-based inhibition motifs (ITIMs), they provide little or no insight into molecular mechanisms occurring after C1q engages the cell surface to directly suppress immune cells. We recently demonstrated that C1q’s collagen-like region (CLR) directly engages the collagen immunoreceptor LAIR-1 (CD305) on monocytes to phosphorylate cytoplasmic LAIR-1 inhibitory motifs (ITIM) and restrict monocyte/monocyte-derived dendritic cell (mono-DC) differentiation and activation^12^. These studies revealed that C1q engages a plasma membrane receptor with intracellular ITIM activity and provided important insight into molecular mechanisms of C1q control over monocyte/DCs. Because of the prevalence of gC1q interactions, we surmised that globular heads of C1q might still be available to bind molecules on the plasma membrane and contribute to C1q’s control over monocyte activity.

CD33 (Siglec-3), another inhibitory immunoreceptor, is a member of the sialic acid immunoglobulin (Ig)-like lectin (siglec) group of proteins functionally categorized by their ability to promote sialic acid dependent cell adhesion. CD33 is described as the smallest siglec member. It features one extracellular V-like (V) domain responsible for recognition of sialic acid, one extracellular Ig C2-like (C2) domain with unknown function, and in the cytoplasm, one ITIM and one ITIM-like sequence^14^. Two isoforms of CD33 exist in humans, one containing the full length protein (CD33M); the other lacking the V domain (CD33m). Both are expressed on the cell membrane, however, the biological function of CD33m remains elusive^15, 16^. Unlike LAIR-1 which is ubiquitously expressed on hematopoietic cells, CD33 is restricted to the myeloid cell compartment^17^. Both LAIR-1 and CD33 are highly expressed on freshly isolated blood monocytes^14, 15, 18^ and activation of CD33 ITIM also restricts monocyte/mono-DC activation and differentiation^17, 19–21^. Phosphorylation of ITIM sequences on CD33’s cytoplasmic tail occurs when CD33 extracellular motifs are cross-linked with anti-CD33 Abs or chemicals, however, natural ligands remain poorly categorized^22^.

Given that gC1q binds to C2-like motifs on molecules other than Ig^1, 23, 24^ and that CD33 contains a C2-like (CD33C2) domain that may be recognized by gC1q, we hypothesized that C1q binds to CD33. Because different immunoreceptors may cooperate to promote inhibitory action^25, 26^, we also hypothesized that C1q crosslinking of CD33 and LAIR-1 on the cell surface would yield activation of both CD33 and LAIR-1 ITIMs. Masking of CD33C2 domains has been described in CD33^+^ cell lines presumably as a consequence of *cis* interactions between membrane associated sialic acid and sialic acid ligands^15^. We therefore, considered whether masking of CD33C2 domains by cell surface sialylation would affect C1q binding.

Genetic or acquired C1q deficiency in systemic lupus erythematosus (SLE), was instrumental in exposing C1q’s complement-independent, tolerogenic role^27^. Nothwithstanding, much remains to be clarified regarding molecular mechanisms involving C1q in this setting. Based on the assumption that accelerated activation and differentiation of SLE monocytes^28–30^ might be associated with flawed LAIR-1 and/or CD33 activity, we investigated patterns of LAIR-1 and CD33 expression on SLE monocytes. Finally, we studied whether CD33 and LAIR-1 expression is regulated by cytokines promoting the growth of anti-inflammatory myelomonocytes versus mono-DCs.

Our studies establish that C1q is a ligand for CD33. Moreover, our findings substantiate that C1q specifically binds to CD33C2 domains and that gC1q and C1qCLR engage CD33 and LAIR-1, respectively, to yield molecular partnering. The lack of LAIR-1 and CD33 expression on SLE monocytes, along with the frequent abnormalities related to C1q in SLE^27^, suggest that C1q/CD33/LAIR-1 inhibitory networks are disrupted in SLE. Masking of CD33C2 domains by sialic acid impedes C1q binding, revealing a possible mechanism for modulating C1q-CD33 inhibitory activity. Biological control of LAIR-1 and CD33 expression was further indicated by the observation that the anti-inflammatory cytokine M-CSF, but not DC growth factors (GM-CSF/IL-4), sustained CD33/LAIR-1 expression on both healthy and SLE cells.

## Results

### Evidence for a C1q-CD33 interaction

**Figure 1:**
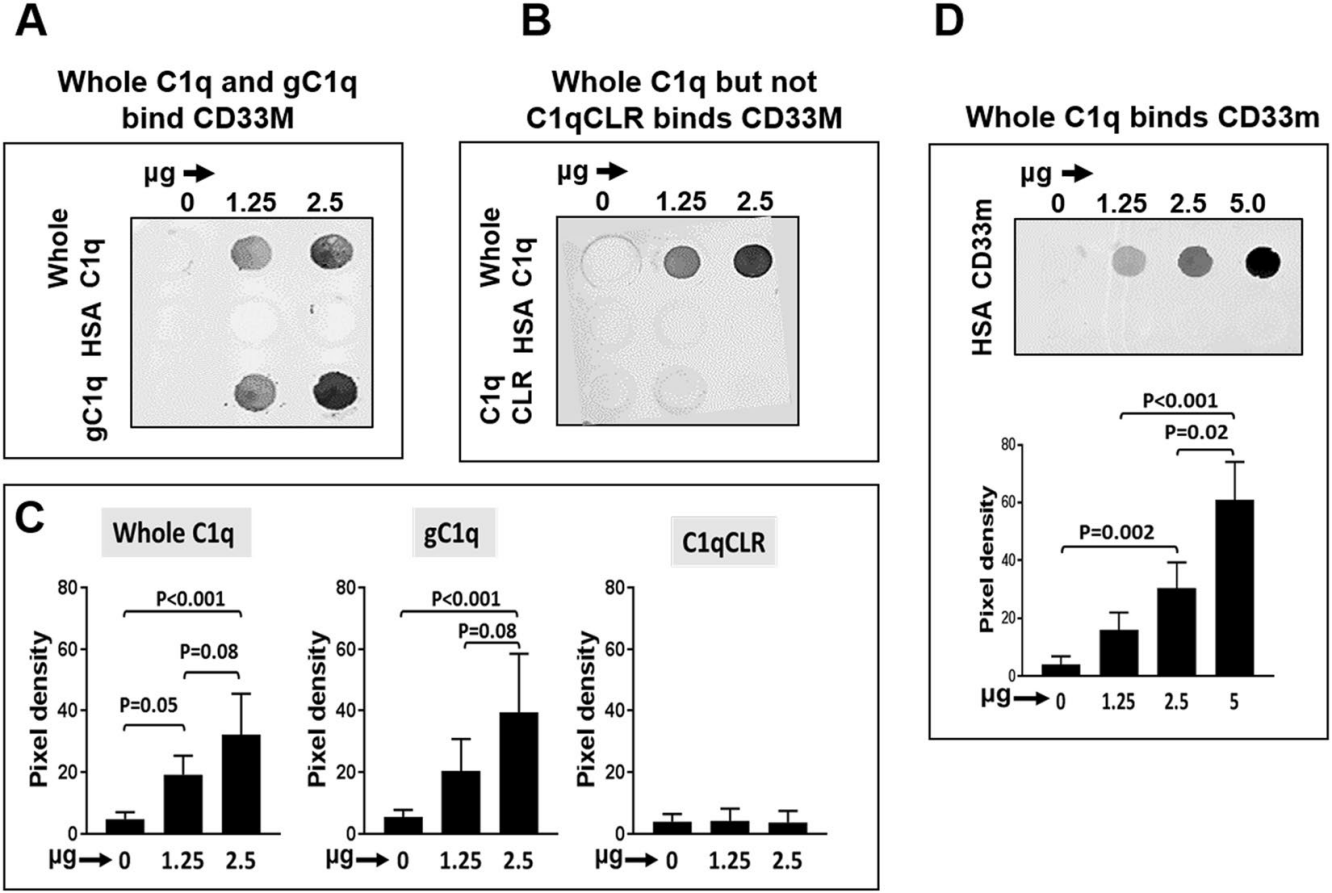
Evidence for domain-specific and concentration dependent associations between C1q and CD33 using purified proteins in slot blot assays. Unlabeled proteins were immobilized on membranes; biotin labeled proteins were in solution. (**A–C**) Dose related binding of immobilized whole C1q and gC1q, but not CLR, to biotin-CD33M (5 ug/ml). (**D**) Biotin-whole C1q (10 ug/ml) binds to immobilized CD33m (lacking the extracellular V-like domain and expressing the C2-like domain) in a dose specific manner. (HSA = human serum albumin control showing no reactivity. Bound proteins were detected using streptavidin conjugated Infrared 800 (LI-COR). Pixel density was determined by densitometry. **(C**,**D)** Bar graphs represent the mean ± SE of pooled data; one-way ANOVA followed by Tukey’s pairwise multiple comparison was used to determine significance. N ≥ 3.

We tested whole C1q and C1q fragments for binding to CD33 isoforms (CD33M, CD33m) in both protein and cell based assays. In Fig. 1, we compared purified protein-protein interactions between whole C1q, gC1q, or C1qCLR and the CD33 M/m isoforms in slot blot assays. In support of direct C1q-CD33 interactions, whole C1q associated with CD33M in a dose dependent fashion whereas binding to a control protein (HSA) was minimal at all doses tested (Fig. 1A,B Supplementary Fig. S1A). While CD33M interacted with gC1q, there was no interaction of CD33M with C1qCLR, even when higher concentrations were used (Fig. 1A,B). Thus, C1q may bind CD33 through its globular head, independent of the CLR. Demonstrating C1q’s direct interaction with the CD33C2 domain, concentration dependent associations were observed between whole C1q and the splice variant CD33m expressing a C2-like domain and lacking the V-like region (Fig. 1D). Direct binding of gC1q, but not C1qCLR to CD33M was also observed using plasmon resonance (Supplementary Fig. S1B).

**Figure 2:**
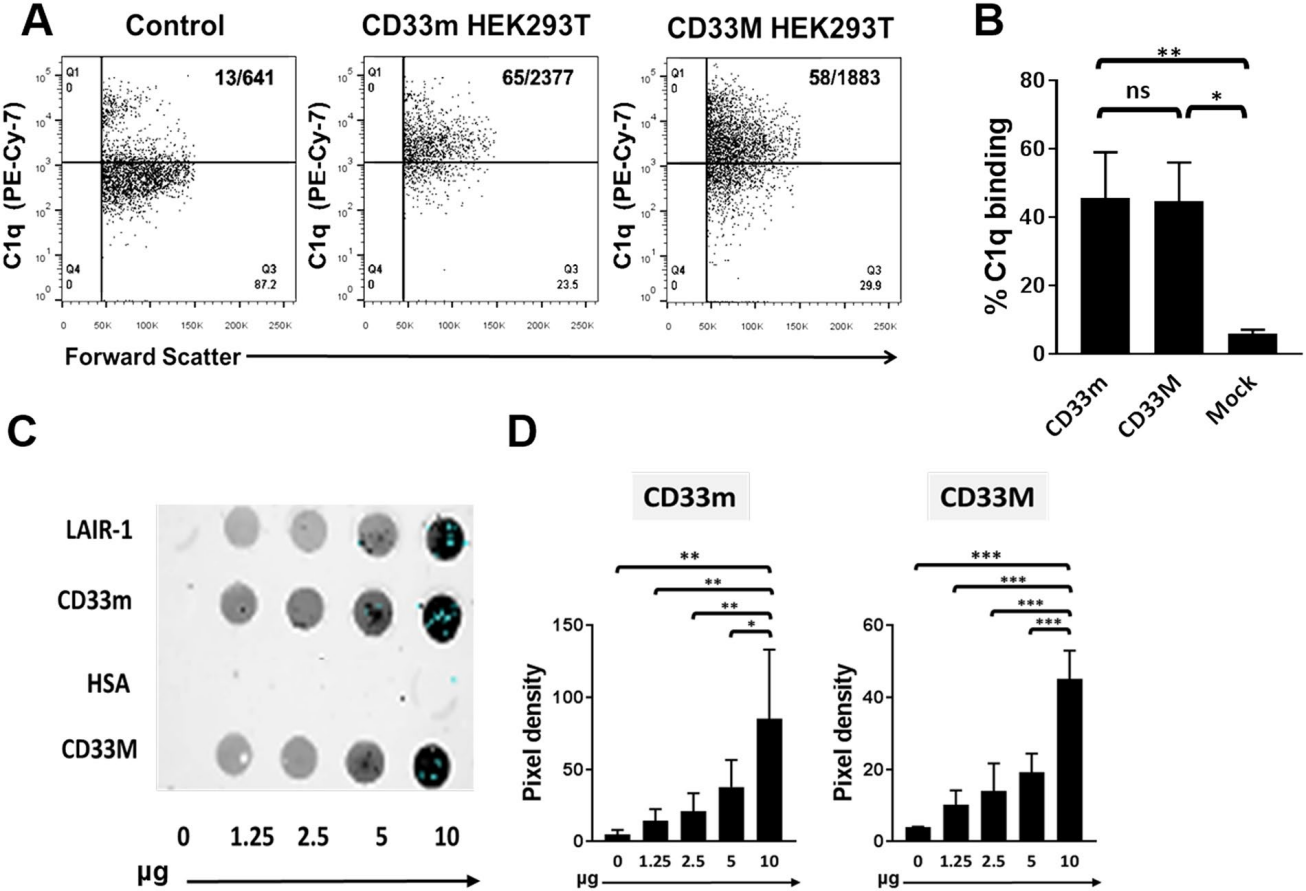
C1q binds to CD33M and CD33m isoforms on the cell surface. (**A**) A representative flow cytometry plot showing binding of biotin C1q (20 ug/ml) to HEK293T cells transfected with plasmids encoding CD33m and CD33M and control mock plasmid. Cells were incubated with C1q as outlined in Materials and Methods. Numbers in graphs = % positive/MFI. Debris/dying cells was excluded on the basis of forward and side scatter. (**B**) Pooled flow cytometry data illustrating binding of C1q (at comparable levels) on the surface of CD33m/M transfected HEK293T cells and a lack of C1q binding on mock transfected cells. For A and B, N = 3. (**C**) Representative slot blot showing that C1q binds to cell-associated CD33m, CD33M or LAIR-1 in a dose dependent manner. Protein prepared from whole cell lysates (HEK293T cells transfected with plasmid encoding CD33m, CD33M or LAIR-1) was immobilized on membranes at increasing doses; biotin-whole C1q was in solution at 10 ug/ml. HSA = human serum albumin control. Protein concentration was calculated by the BCA method. Bound proteins were detected using streptavidin conjugated Infrared 800 (LI-COR). (**D**) Pooled data analyses illustrating dose related interactions between biotin-C1q and cell-associated CD33m or CD33M represented in (**C)**. (**B**,**D**), the mean ± SE of pooled data is shown; for all proteins and doses, N = 3–5. *P =  < 0.05, **P = <0.01, ***P < 0.001. One-way ANOVA followed by Tukey’s pairwise multiple comparison was used.

To corroborate C1q binding to CD33 on the cell surface and the involvement of C2 domains in the C1q-CD33 interaction, HEK293T cells were transfected with cDNA encoding full length CD33M (~67 kD) or CD33m (~31 kD)^15, 31^. Flow cytometry-assisted analysis using CD33 domain-directed antibodies (HIM3–4 mAb specific to a CD33C2 epitope; WM53 mAb specific to a CD33V epitope), along with SDS-PAGE immunoblots, confirmed expression of each protein in transfected cells only (Supplementary Fig. S1C,D). As shown in Fig. 2A,B, C1q bound to CD33m and CD33M transfected HEK293T cells; binding of C1q to mock (control) cDNA transfected or untransfected cells was negligible. Furthermore, cell lysates prepared from transfected cells revealed the capacity of whole C1q to bind cell-associated CD33m and CD33M in concentration dependent fashion (Fig. 2C,D). Collectively, these data suggest that biologically relevant C1q-CD33 interactions may occur through C1q’s globular head, independent of the CLR and the CD33 sialic acid binding V-like region. As previously reported by us^12^, C1q also bound LAIR-1 in a dose dependent manner (Fig. 2C).

### C1q-triggered phosphorylation of CD33 and LAIR-1 ITIM involves C1q-CD33/LAIR-1 partnering in human monocytes

**Figure 3:**
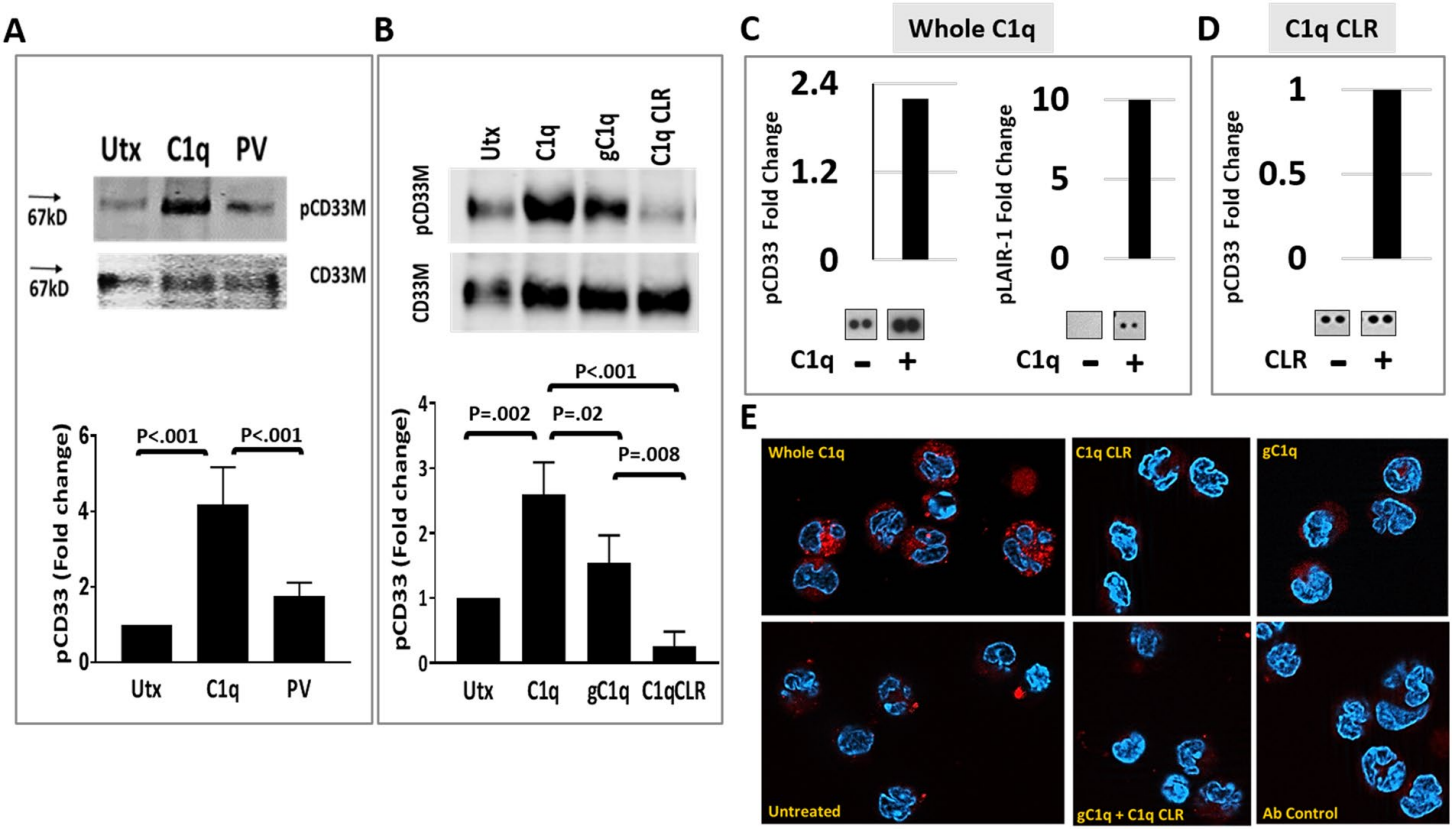
C1q triggers CD33 ITIM phosphorylation and CD33-LAIR-1 physical associations in human monocytes. Monocytes were either untreated (utx), treated with whole C1q, gC1q, C1q CLR or pervanadate (PV) as detailed in Materials and Methods. (**A**,**B**) Immunoblot analyses showing increased phosphorylated (p) CD33 after treatment with C1q and gC1q, but not C1q CLR. CD33 was immunoprecipitated with anti-CD33M antibody; tyrosine phosphorylation of CD33 (pCD33M) was detected with anti-phosphotyrosine (4G10) antibody. The membrane was stripped and re-probed with anti-CD33 WM53 antibody (CD33M). Arrows denote molecular weight of 67kD. Bar graphs denote pooled data expressed as fold change relative to total CD33 and corresponding statistical analyses. One-way ANOVA with post-hoc Tukey multiple comparisons was used to determine significance. N = 4 for A; N = 3 for B. (**C**) The addition of whole C1q (20 ug/ml) prompts concurrent increases in pLAIR-1 and pCD33 in a phospho-immunoreceptor array. (**D**) C1q CLR “tails” (20 ug/ml) do not elicit increases in pCD33M in the phospho-immunoreceptor array. Bar graphs represent fold change (plus/minus C1q), calculated from the mean pixel density of duplicates, as determined by densitometry analysis. Corresponding C1q minus/plus array data is located below each bar graph in C and D. Data from a typical array are shown. N = 3 for C and D. (**E**) Proximity ligation assay, performed on freshly isolated human monocytes as detailed in materials and methods, showing that whole C1q is required for CD33-LAIR-1 crosslinking. Red fluorescent dots represent molecular associations between CD33 and LAIR-1; blue represents nuclear staining with DAPI. CLR and gC1q represent the C1q collagen tail and globular heads of C1q, respectively. Original magnification = 60X. One of three representative experiments is shown.

Next, we established that C1q stimulates phosphorylation of CD33 ITIM in freshly isolated human blood monocytes (Fig. 3). Western blot analyses of proteins immunoprecipitated with anti-CD33 mAb demonstrated that C1q increases basal levels of phosphorylated (p) CD33 (Fig. 3A,B). Whole C1q and gC1q treatment yielded increases in pCD33 (Fig. 3B) indicating that CD33 inhibitory signaling may be accomplished via C1q’s globular heads. The higher levels of pCD33 achieved with whole C1q versus gC1q are in line with increased binding sites on the multivalent whole C1q. While CLR induces pLAIR-1^12^, it did not increase levels of pCD33 ITIM, as demonstrated in both the immunoprecipitation assay (Fig. 3B) and phosphoarray (Fig. 3D). These findings are consistent with the lack of physical interaction between CLR and CD33 (Fig. 1B). C1q treatment increased basal levels of pCD33 (2–5 fold increases C1q versus controls, n ≥ 6) in both the western blot and phosphoarray. Thus, in addition to inducing pLAIR-1, a direct biological effect of C1q on monocyte function may be to activate CD33 inhibitory signaling.

Indicative of CD33-LAIR-1 associated biological activity, whole C1q induced concurrent phosphorylation of CD33 and LAIR-1 ITIMs in the phospho-immunoreceptor array (Fig. 3C). Although activation of CD33 ITIM has been shown to inhibit CD64 (FcγRI) related ITAM activity^17^, it has also been reported that tyrosine phosphorylation of CD33 is not dependent on CD32a (FcγRIIa) or CD64 ITAM activation^26^. In agreement with a lack of CD32a ITAM involvement in pCD33, we noted little or no change in phosphorylation of CD32 after monocytes were treated with C1q (Supplementary Fig. S1E).

In order to further substantiate associated biological activity we studied whether C1q physically crosslinks CD33 and LAIR-1 in a proximity ligation assay (PLA) (Fig. 3E). In this assay, the pairing of complementary DNA sequences (attached to secondary antibody) and subsequent *in situ* amplification of DNA occurs only when CD33 and LAIR-1 are in close proximity (<40 nm). Red fluorescent dots denote co-ligation and the number of dots is strictly proportional to the number of C1q-CD33-LAIR-1 interactions. Monocytes treated with whole C1q and both anti-LAIR-1 and anti-CD33 Abs produced strong fluorescent signals. In contrast, untreated monocytes or monocytes incubated with either anti-LAIR-1 or anti-CD33 Ab alone displayed fewer red fluorescent dots. Furthermore, gC1q and CLR, used alone or in combination, failed to produce a strong fluorescent signal. These results establish that whole C1q acts as a molecular bridge between LAIR-1 and CD33 on the monocyte surface and are consistent with the concept that the globular heads of C1q bind CD33 while its collagen tail engages LAIR-1. They also suggest that simultaneous phosphorylation of CD33 and LAIR-1 ITIM (Fig. 3C) may be a functional consequence of C1q-facilitated complexing of CD33 and LAIR-1 at the monocyte surface, as hypothesized in Supplementary Fig. S2.

### Biological control of C1q/CD33/LAIR-1 processes

**Figure 4:**
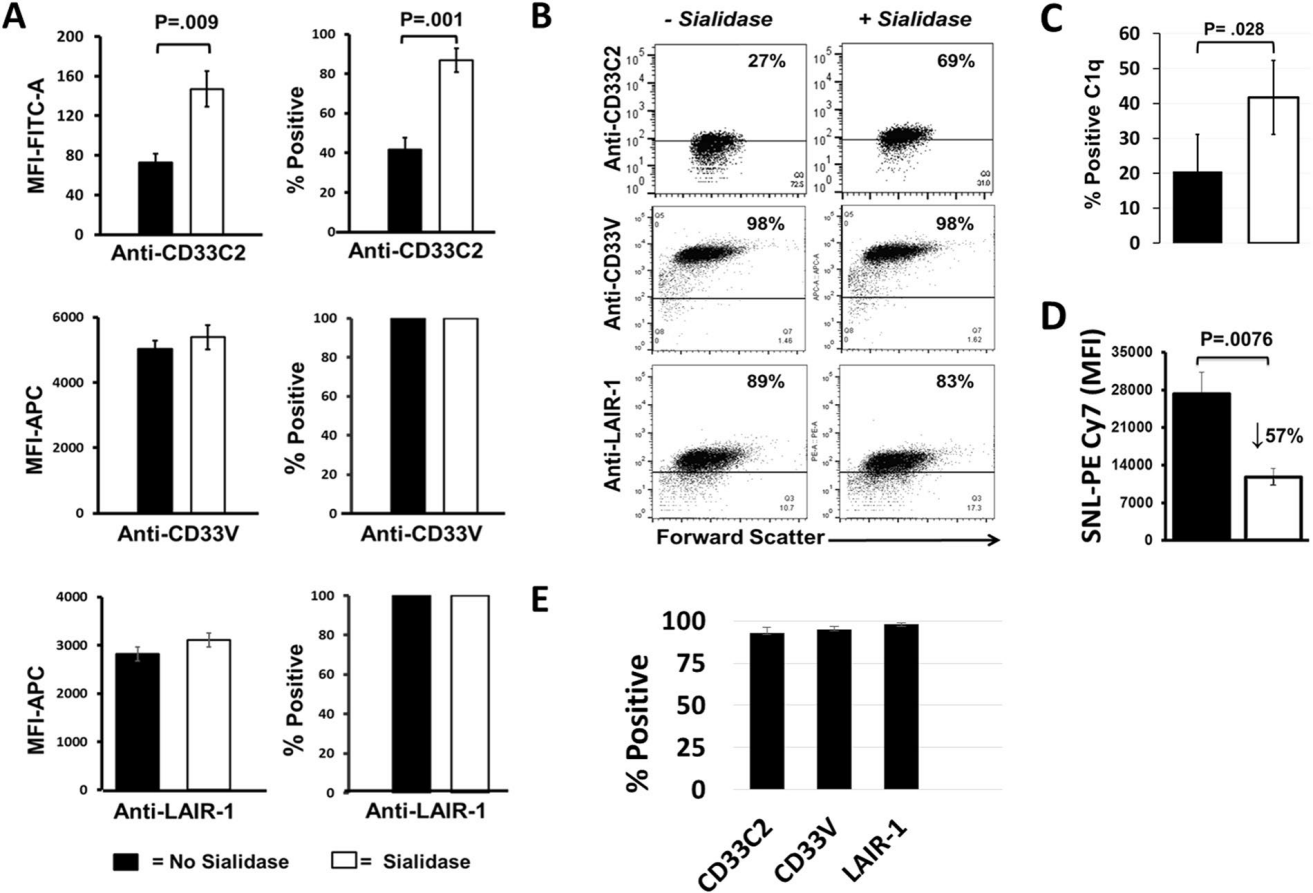
Masking of CD33C2 domains impedes C1q binding on the cell surface. (**A**,**B**) Increased detection of the CD33C2, but not the CD33V domain or LAIR-1, occurs after sialidase treatment of THP-1 cells. Cells were treated with sialidase (20 mU/10^6^ cells) for 60 min at 37 °C in a humidified incubator, resuspended twice in PBS, then in staining buffer for detection of CD33C2 domains (HIM3-4), CD33V domains (WM53) and LAIR-1. A = Pooled data, N ≥ 4. B = Representative experiment from A; gates were set according to isotype Ab controls. (**C**) Unmasking of CD33C2 domains with sialidase correlates with increased binding of C1q on THP-1 cells; N = 3–4. (**D**) Reduced levels of sialic acid on the THP-1 surface after sialidase treatment as revealed by decreased binding to Sambucus *nigra* lectin (SNL) (5 ug/ml). N = 5. (**E**) Pooled flow cytometry data showing highly expressed CD33C2 and CD33V domains and LAIR-1 on freshly isolated normal monocytes. Results represent mean +/− SEM. N = 3–4. For (**A**,**C**,**D**) solid bars = no sialidase; open bars = sialidase. Two-tailed Student’s t test was used to determine significance.

While masking of siglecs, in particular the B cell associated CD22 receptor, by sialic acid has been associated with control of B cell activation^14^, functional consequences of masking of CD33C2 domains are poorly understood. Detection of the CD33C2 domain, but not the CD33V2 domain, was markedly reduced on THP-1 myelomonocytic cells (Fig. 4A,B). This was in marked contrast to freshly isolated monocytes from healthy individuals where both CD33C2 and CD33V2 were detected on the majority of cells (Fig. 4E). In agreement with other reports testing CD33C2 domains on U937 leukemic cells^15^, we found that sialylation interferes with detection of CD33C2 domains on THP-1 cells. As shown in Fig. 4A,B, sialidase treatment produced significantly increased detection of CD33C2, as revealed with the C2 domain specific HIM3-4 mAb (Fig. 4A,B). Given the interaction of C1q with CD33m, we asked whether masking of CD33C2 domains on THP-1 cells would interfere with C1q binding. Whereas only a minority (~20%) of untreated THP-1 cells bound C1q in a dose dependent manner (Fig. 4C; Supplementary Fig. S3A), sialidase treatment of THP-1 cells at least doubled the number of cells binding to C1q (Fig. 4C). As previously reported for U937 cells, no difference in detection of CD33V epitopes using WM53 mAb occurred after sialidase treatment (Fig. 4A,B). Levels of LAIR-1 (Fig. 4A,B) or other putative gC1q receptors (p33; RAGE) were also not altered by sialidase treatment (Supplementary Fig. S3B–D). Thus, the ability of C1q to bind CD33C2 domains may be specifically compromised by sialylation.

### Patterns of CD33 and LAIR-1 expression are similarly altered on mono-DCs and SLE blood myelomonocytes

**Figure 5:**
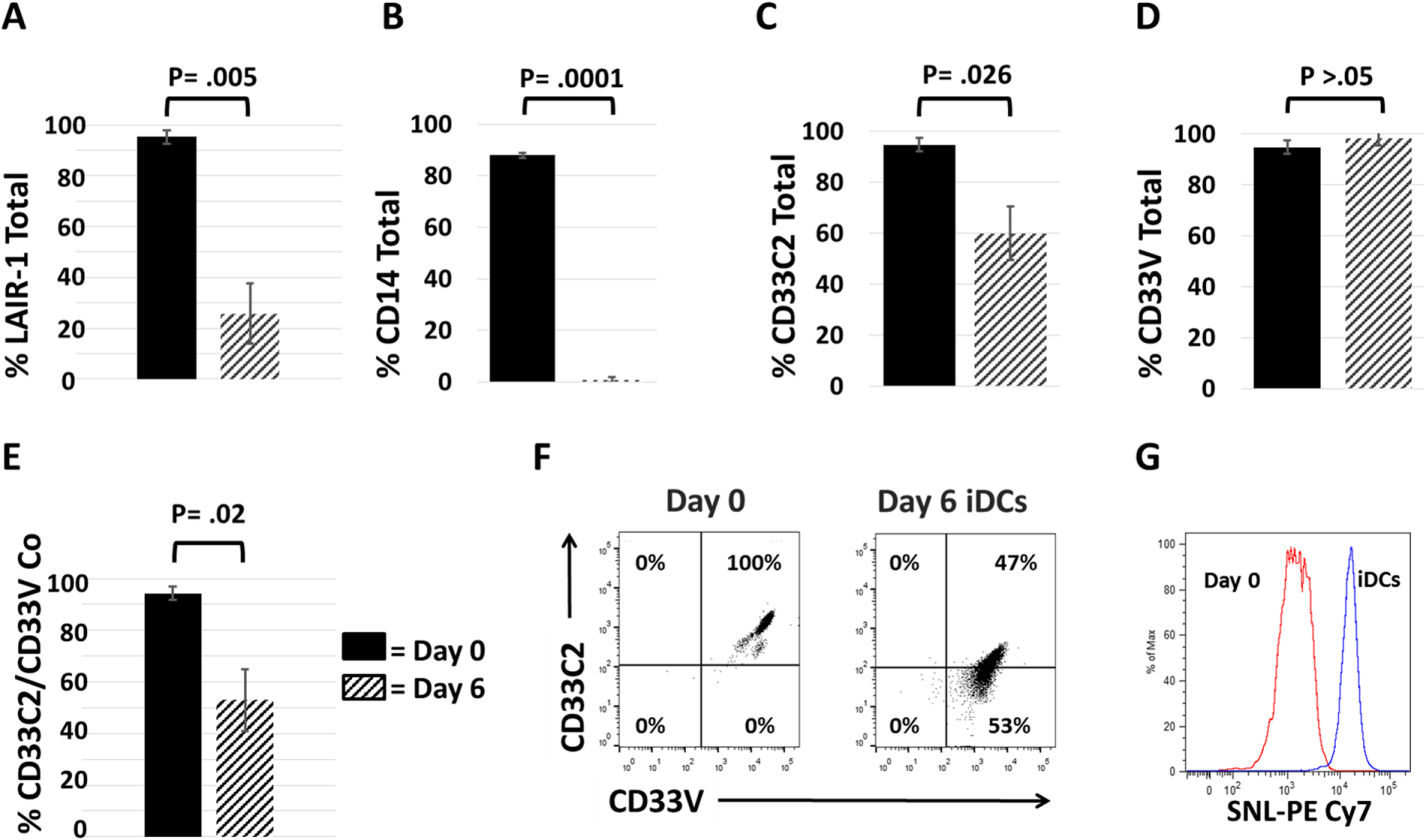
Maturing DCs exhibit decreases in LAIR-1 and CD14 along with a reduced ability to detect CD33C2 epitopes. (**A**,**B**) Decreased expression of LAIR-1 and CD14 on DCs. (**C**,**D**) Pooled flow cytometry data showing CD33C2 domains (detected with mAb HIM3-4) and CD33V domains (detected with mAb WM53) on freshly isolated monocytes and DCs obtained after 5–6 days of culture with 50 U/ml rGM-CSF and 50 ng/ml rIL-4. (**E**) Pooled data showing co-detection of CD33C2 and CD33V domains on fresh monocytes and day 6 DCs. (**F**) Representative dot plot comparing co-detection of CD33C2/V epitopes on freshly isolated monocytes and day 6 DCs; gates were set based on isotype controls. **(G**) Increased levels of sialic acid on immature DCs detected with Sambucus *nigra* lectin (SNL) linked to PECy7. For (**A**–**E**), N = 3–5; for (G), N = 2. Two-tailed Student’s t test was used to determine significance. Solid bars represent day 0; hatched bars represent day 6.

Taking into consideration that levels of membrane-associated sialic acid increase during *in vitro* mono-DC differentiation^32^, the elevated sialic acid levels in SLE blood^33^, and that a strong pro-inflammatory environment exists in both settings, we investigated patterns of CD33/LAIR-1 expression on mono-DCs and SLE blood myelomonocytes. While the distribution of CD33 on SLE cells and DCs has been studied, to our knowledge, parallel studies comparing detection of CD33C2 and CD33V domains on these cells has not been performed. As previously shown by us and others^12, 34^, LAIR-1 expression was reduced on mono-DCs on day 6 compared to freshly isolated monocytes obtained from healthy subjects (Fig. 5A). Nearly absent levels of CD14 signified commitment to the DC lineage (Fig. 5B)^12^. Corresponding assessment of CD33 expression revealed that detection of CD33C2 domains, but not CD33V2 domains was significantly reduced with DC differentiation (Fig. 5C,D). Dual label analysis also showed that whereas CD33C2 and CD33V domains are co-detected on the majority of freshly isolated monocytes, detection of CD33C2 domains occurred on only a fraction of CD33V domain positive DCs (Fig. 5E,F). By day 3, mono-DC differentiation was accompanied by increased surface sialylation (Fig. 5G). Thus, like THP-1 cells, masking of CD33C2 domains by sialic acid may occur on differentiating DCs. In support of this concept, our preliminary studies reveal increased detection of CD33C2 domains after sialidase treatment of immature DCs (Supplementary Fig. S3E).

**Figure 6:**
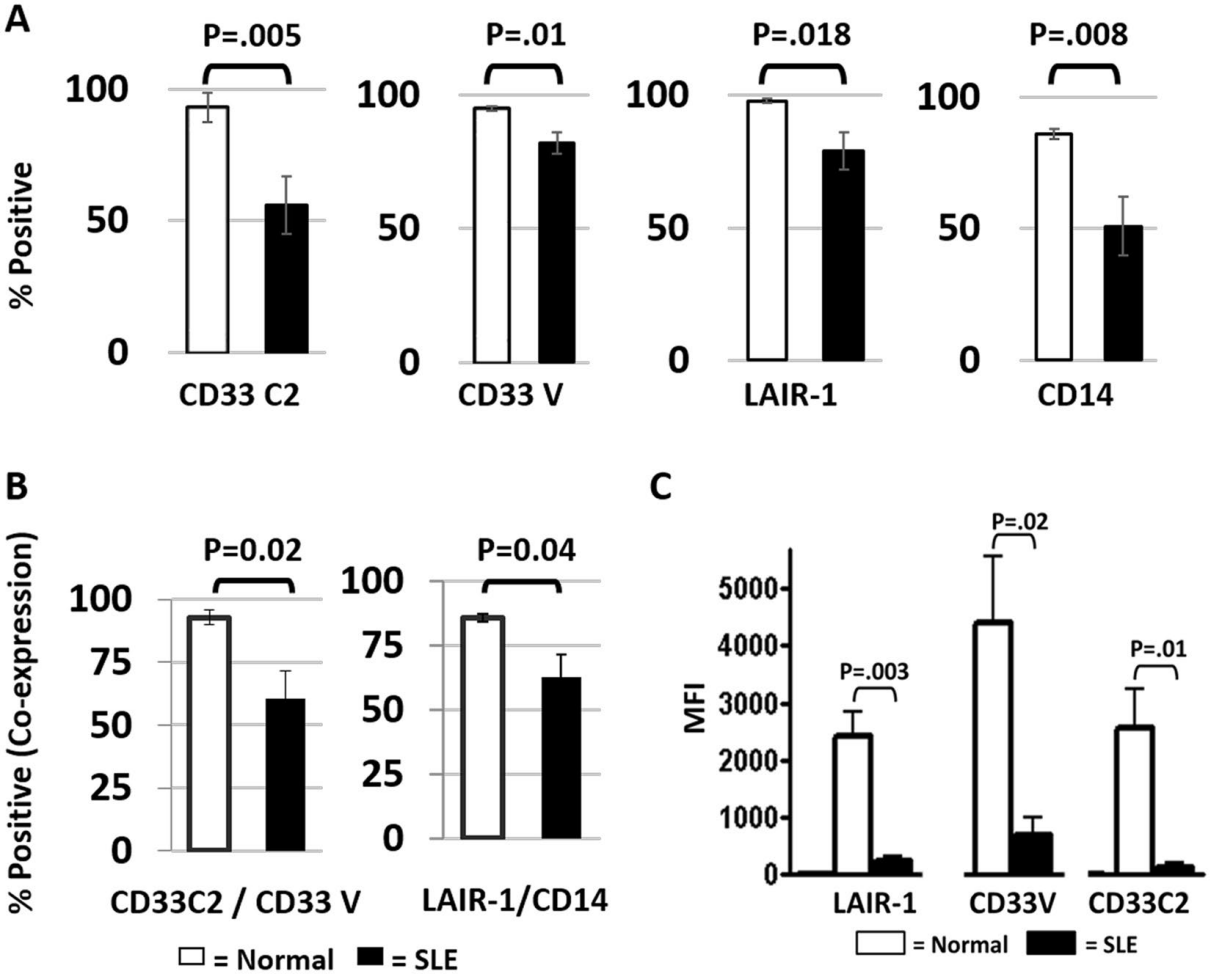
Altered patterns of CD33C2/V and LAIR-1 expression on freshly isolated SLE versus normal blood monocytes. (**A**,**B**) Decreased CD33C2/CD33V, LAIR-1, CD14, on SLE blood myelomonocytes vs healthy monocytes as revealed by percent positive (**C**) Decreased LAIR-1, CD33C2/CD33V domains on SLE blood monocytes vs healthy monocytes as revealed by Mean Fluorescence Intensity (MFI). In (**A**–**C**) open bars represent normal (NL) monocytes; solid bars represent SLE myelomonocytes. Results represent the mean +/− SEM of pooled flow cytometry data; two-tailed Student’s t test (**A–C**) was used to determine significance. For (**A**,**B**); N = 9 for CD33C2 and CD33V; N = 8 for LAIR-1 and CD14 for both NL and SLE. For (**C**); N = 3 for both normal and SLE blood. Gate settings for flow analysis excluded debris and dead cells and are described in Supplementary Fig. S4.

As reported by others^29, 30^, we found circulating SLE myelomonocytes to be lacking in CD14 expression and severely altered in phenotype/morphology (Fig. 6; Supplementary Fig. S4). Analysis of CD33 and LAIR-1 expression on SLE myelomonocytes revealed that detection of CD33C2/V domains and LAIR-1 was reduced compared to healthy monocytes (Fig. 6). Despite decreases related to the CD33V domain, most (>70%) of the cells were still positive (Fig. 6A). The CD33C2 domain was detected on fewer SLE cells and, reminiscent of immature DCs (Fig. 5E), the CD33C2 domain was not detected on many CD33V domain-expressing cells (Fig. 6B). Also consistent with a mono-DC like phenotype we noted decreased levels of CD14 and LAIR-1 in the SLE cohort we studied (Fig. 6A,B). Cells co-expressing LAIR-1 and CD33C2 domains were also lacking in SLE (Supplementary Fig. S4C). Based on the similar patterns of LAIR-1 and CD33C2 domains it is tempting to speculate shared regulatory processes, which, aside from inflammatory cytokines, could be influenced by increased levels of sialic acid. Whether masking of CD33C2 domains or lack of LAIR-1 would interfere with C1q’s ability to suppress myelomonocytes/DCs remains to be demonstrated.

### M-CSF sustains coupled LAIR-1 and CD33 expression

**Figure 7:**
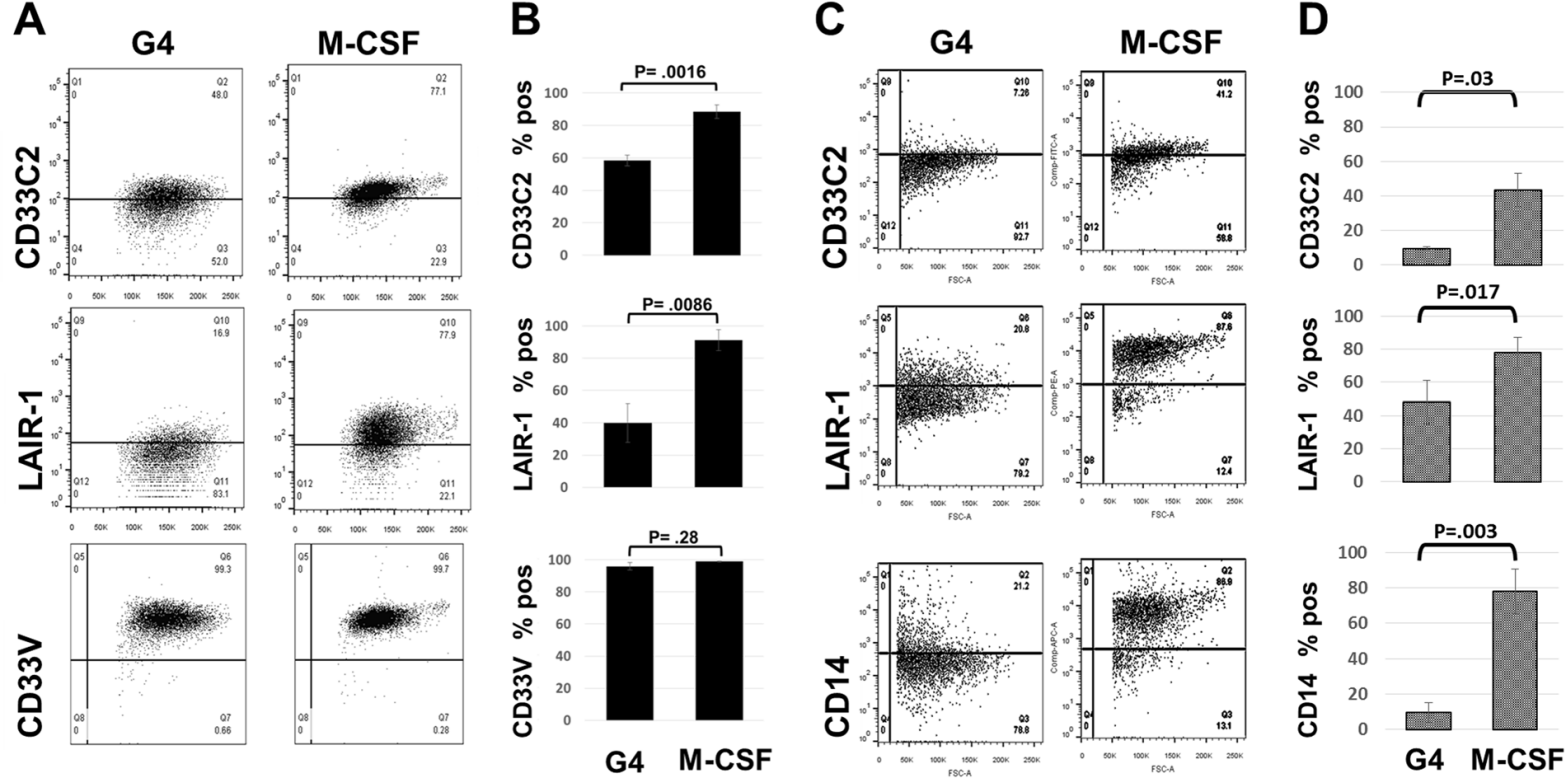
Myeloid cytokines distinctly control the coupled expression of CD33C2 and LAIR-1 in healthy and SLE cells. Freshly isolated adherent mononuclear cells from healthy subjects (**A**,**B**) and SLE patients (**C**,**D**) were cultured in parallel with either GM-CSF/IL-4 (G4) or M-CSF for 5 days, stained with anti-LAIR-1, CD33C2, CD33V antibodies and then analyzed by flow cytometry. (**A**,**C**): dot plots represent fluorescence versus forward light scatter patterns for a typical experiment; (**B**,**D**) represent the mean +/− SEM of pooled flow cytometry data. Two-tailed Student’s t test was used to determine significance. For normals, G4 vs M-CSF; N = 5 for CD33C2, N = 6 for CD33V, N = 4 for LAIR-1. For SLE, G4 vs M-CSF; N = 4 for CD33C2, LAIR-1; N = 3 for CD14.

In order to gain further insight into the biologic control of LAIR-1 and CD33, we compared in parallel, the LAIR-1/CD33 receptor profile of cells from healthy and SLE subjects cultured for 5 days under anti-inflammatory conditions with M-CSF versus pro-inflammatory conditions using DC growth cytokines (GM-CSF + IL-4) (Fig. 7). As expected for healthy subjects, LAIR-1 expression was reduced in mono-DC cultures (Fig. 7A,B) versus baseline values on day 0 (~95%, Fig. 5A). In marked contrast, with M-CSF, detection of LAIR-1, CD33C2, and CD33V, and CD14 resembled the high baseline levels found in healthy monocytes (Fig. 5). Detection of CD33V domains did not differ between cells treated with M-CSF or GM-CSF + IL-4. Notably, similar analyses of SLE cells (Fig. 7C,D) also yielded increases in LAIR-1, CD33C2 and CD14 with M-CSF versus mono-DC cytokines. Levels of CD14 achieved with M-CSF resembled the high baseline values noted in healthy subjects. Suggestive of greater resistance to M-CSF effects, levels of CD33C2 in the M-CSF treated SLE cells did not reach the baseline levels observed in healthy monocytes, although they were higher than the GM-CSF + IL-4 treated SLE cells. Thus, whereas pro-inflammatory cytokines reduce detection of both LAIR-1 and CD33C2, M-CSF (a prototype anti-inflammatory cytokine normally present in steady state) sustains coordinate expression of LAIR-1 and CD33C2.

## Discussion

Growing evidence establishes that C1q’s role in modulating myeloid cell activity includes its ability to prevent unwarranted activation of circulating monocytes and the development of mono-DCs^8, 10, 12, 35^. Previously, we outlined that the ability of C1q to directly inhibit monocyte activity and mono-DC differentiation occurs via the immunoreceptor LAIR-1^12^. Here, we expand on those findings and substantiate that while C1q’s collagen tail engages LAIR-1, C1q’s globular head will interact with CD33 to promote phosphorylation of CD33 ITIM and a C1q/CD33/LAIR-1 inhibitory complex. Our results are in agreement with others that crosslinking of CD33 at the cell surface triggers tyrosine phosphorylation of CD33 ITIMs in myeloid cells^17, 26^, and to our knowledge, represent the first identification of a natural ligand triggering pCD33.The blocking effects of sialic acid on C1q binding also suggest a process for regulating monocyte activity. Biological control of LAIR-1 and CD33 expression was further indicated by the observation that the anti-inflammatory cytokine M-CSF, but not DC growth factors (GM-CSF/IL-4), sustained CD33/LAIR-1 expression on both healthy and SLE cells. Finally, the lack of LAIR-1 and CD33 expression on circulating SLE myelomonocytes, along with the frequent abnormalities related to C1q in SLE^27^, are consistent with the idea that C1q/CD33/LAIR-1 tolerogenic networks are disrupted in SLE.

Beyond substantiating that C1q-CD33 binding on monocytes promotes phosphorylation of CD33 ITIM, C1q’s ability to interact with CD33m in protein assays and CD33m on the surface of transfected HEK293T cells implies a biological role for CD33C2 domains and the naturally occurring CD33m isoform. Whole C1q and gC1q, but not CLR, interacted with CD33 suggesting direct associations between gC1q and CD33C2 regions. Because CD33m lacks the V domain conferring sialic acid binding specificity, our results show that binding of CD33 to C1q may occur independently of its sialic acid binding domain. Considering that sialic acid is exclusively located in the C1q globular region^36^ we cannot discount the possibility that interactions between the CD33V regions with sialic acid on gC1q also occur. It is also possible that CD33V domains, by engaging negatively charged sialic acid bearing molecules will recruit the highly positively charged C1q and therefore contribute to the stability of C1q/CD33 complexes on the cell surface.

Due to its multimeric nature, C1q may serve not only as a molecular bridge between LAIR-1 and CD33 on the cell surface, but also as a signal amplification molecule aimed at securing immune quiescence. Previously, we noted that even though CLR fragments interact with LAIR-1 to activate LAIR-1 ITIM, stronger interactions occur between whole C1q and LAIR-1^12^. Thus, it is possible that anchoring C1q’s globular region to the cell surface via CD33 would increase the number of LAIR-1 receptors engaging the multiple collagen repeats available on CLR for enhanced cross-linking and stronger inhibitory action. Also contributing to the enhancement of inhibitory signaling would be the ability of C1q’s globular heads to engage distinct CD33C2 domains on the cell surface. Hypothetical C1q-CD33-LAIR-1 interactions are depicted in Supplementary Fig. S2.

Others^37, 38^ studying modulation of cell activation via CD33 and CD22 have demonstrated both *cis* and *trans* activation of these receptors. Interestingly, multivalent ligands acting in *trans*, and predicted to displace *cis* interactions involving CD22, lead to suppressed B cell activity^38^. In our studies, preliminary findings implied reduced CD14-CD33 *cis* interactions on monocytes after treatment with whole C1q in the PLA (Supplementary Fig. S1F). Given the multivalent nature of whole C1q and that sialylated gC1q may interact with both CD33C2 and V domains, it is conceivable that binding of C1q’s globular region to CD33 in *trans* may lead to displacement of *cis* interactions between CD33 and CD14 in favor of CD33-LAIR-1 partnering. In any event, because C1q is normally present in the circulation during steady state^39^, and C1q has been demonstrated on the surface of monocytes^8^, we contemplate that some circulating C1q constitutively binds CD33 to promote steady state tolerogenic activities.

Whether CD14-CD33 interactions contribute to the constitutive pCD33 activity in human monocytes noted by us (Fig. 3) and others^37^ still needs to be addressed. It has been reported that *cis* interactions occurring between CD33 and sialylated CD14 prevent LPS-mediated toll- like receptor4 (TLR4) signaling, independently of CD33 ITIM^40^. Thus, CD14-CD33 partnering may represent a distinct, complementary process for regulating inflammatory responses. Specifically, while LPS-CD14-CD33 associations would suppress CD14 dependent TLR4 signaling, C1q-CD33-LAIR-1 associations would yield ITIM related inhibition. Further work aimed at understanding these complex regulatory processes in normal and abnormal physiology would involve studying the contribution of LPS-CD14-CD33 and C1q-CD33-LAIR-1 partnering in distinct biological settings. Consideration must be given to the stage of cell differentiation and the contribution of other C1q ligands such as RAGE and CD91 in determining the level of inhibition ultimately achieved.

Even though CD33 is a major marker on myeloid cells, domain specific functions of CD33 and the role of alternate splice variants of CD33 in normal and abnormal physiology remains elusive^41, 42^. In contrast to normal blood monocytes where both CD33C2 and CD33V domains and LAIR-1 were readily detected by antibodies, we observed less binding of CD33C2 domains, but not CD33V domains or LAIR-1, on THP-1 cells. Substantiating that the ability to detect CD33C2 domain-C1q interactions may be compromised by cell surface sialylation, removal of sialic acid on THP-1 cells exposed CD33C2 domains and increased C1q binding. Interestingly in the brain, and in agreement with our findings, C1q binding on the cell surface may be affected by the sialylated state of the cell and even when C1q is available in the extra cellular space, it may not always be engaged on the cell surface^43^. Since CD33 is abundantly expressed on microglial cells^16, 41, 44^ it is conceivable that masking of CD33C2 domains in the brain influences C1q inhibitory action on microglial cells.

Consistent with release from C1q inhibitory effects, the expression of LAIR-1 is reduced during the differentiation of mono-DCs^12^. We noted that a significantly reduced ability to detect CD33C2, but not CD33V domains was linked to increases in levels of sialic acid in DCs (Fig. 5). Our preliminary studies demonstrating increased detection of CD33C2 domains after sialidase treatment of immature DCs (Supplementary Fig. S3E) are in line with masking of CD33C2 domains by sialic acid. Our findings support the idea that such masking would interfere with C1q’s ability to suppress DC activity. Suggestive that engaging CD33V domains alone does not trigger CD33 ITIM immunomodulatory activity, others^19^ showed that crosslinking of CD33 on the cell surface using CD33V region directed mAbs (including WM53) does not to inhibit the function of mature DCs, which are highly sialylated^32^. Moreover, inhibitory TLR signaling occurring as a result of *cis* interactions between CD33 and CD14 do not appear to be the result of pCD33^40^. Based on these collective observations we advance the idea that CD33 domains on myeloid cells exhibit distinct biological activities; i.e., sialic acid dependent cell interactions via CD33V domains and sialic acid independent interactions, as illustrated by gC1q-CD33C2 binding. Our follow up studies intend to understand the control of these two important functions independently of, and in conjunction with LAIR-1, and, in the context of changes in the C1q receptor profile occurring with cell activation/differentiation^2, 8^.

We anticipate that our studies will help decipher molecular mechanisms contributing to the loss of immune cell quiescence. This includes SLE, which features accelerated monocyte/DC differentiation^28–30^, along with elevated sialic acid levels and decreased C1q levels^27, 33, 45^. Aside from reduced expression of CD33 and LAIR-1, C1q-mediated LAIR-1/CD33 inhibitory activity may be compromised by auto-antibodies directed either to gC1q and/or CLR, both of which have been described in SLE^46–48^. Because LAIR-1 is also abnormally distributed on B cells and plasmacytoid DCs in SLE^49–51^, abnormal C1q function in SLE may be linked to impaired LAIR-1 expression on these cell lineages, independent of CD33.

In strong contrast to GM-CSF + IL4, M-CSF sustained the expression of CD14, LAIR-1, and unmasked CD33C2 domains. Both healthy and SLE cells responded to the effects of M-CSF, indicating a degree of plasticity in the SLE cells and that achievement of a “normalized” inhibitory immunoreceptor profile is possible. While our results demonstrate that coordinate control of LAIR-1 and CD33 expression is possible via M-CSF and mono-DC growth factors, further work is required to address how this may operate in distinct physiological settings.

## Materials and Methods

### Proteins and Reagents

Anti-human (hu) antibodies (Abs) used in flow cytometry, proximity ligation assays (PLA) and immunoprecipitation assays were commercially obtained and included: Anti-LAIR-1 (mAb DX26; BD Pharmingen; rabbit polyclonal, Sigma HPA011155); mAb anti-CD33m (HIM3-4; BD-Pharmingen, Biolegend); mAb anti-CD33M WM53; BD Biosciences, Biolegend); mAb anti-CD14 (BD Pharmingen); rabbit polyclonal anti-CD14 (Abgent Inc.) anti-RAGE (rabbit polyclonal; BIOSS, mAb MM0520-8D11; Abcam); anti-p33 (mAb74.5.2: Santa Cruz Biotech).

Recombinant (r) hu proteins purified from cDNA transfected HEK293 T cells were: full length CD33 (CD33M, Novoprotein Scientific Inc., Short Hills, NJ), CD33m (TP317716, Origene technologies, Rockville, MD), biotin-labeled CD33M (CD3-HB22R; ACRO Biosystems, Bethesda, MD).C1q derived from normal hu serum (Complement Technology Inc., Tyler, TX) was used either as unmodified or biotin labeled, or cleaved into gC1q (collagenase digests) or CLR (pepsin digests) as described^12, 52^. C1q digestion and purity was confirmed by SDS/PAGE. Biotinylated proteins were detected using streptavidin-phycoerythrin (PE)-Cy7 (BD Biosciences); streptavidin-Alexa 488 (Molecular Probes, Invitrogen) or streptavidin-conjugated infrared (LI-COR). For removal of cell surface sialic acid, sialidase (Arthrobacter *ureafaciens*, Roche) was used; verification of sialic acid removal was performed using biotinylated Sambucus *nigra* lectin (SNL, Vector Labs, Burlingame, CA).Other proteins included clinical grade hu serum albumin (HSA, Plasbumin-25,Talecris, Research Triangle Pk, NC), bovine albumin (BSA, Sigma-Aldrich). Protein concentration was determined using the Micro BCA Protein Assay Kit (Thermo Scientific-Pierce).

### Patients, Primary Cells, Cell lines, Transfection

Heparinized blood was obtained from healthy individuals and SLE patients according to institutional guidelines. Experimental protocols were approved by the Feinstein Institute for Medical Research institutional review board. Blood was obtained from 12 SLE patients after written informed consent during routine clinic visits to the Feinstein Institute Lupus Center of Excellence. The cohort was 67% female with stable disease activity as reflected by a mean SLEDAI score of 3 ± 2.92 (range 0–8), normal complement levels; 3 patients displayed elevated anti-dsDNA antibody titers. Additionally, none had active renal disease at the time of the blood draw and all were on prednisone doses of ≤10 mg daily. 58% had a history of renal disease and 92% were taking anti-rheumatic disease modifying drugs, 68% of these were taking mycophenolate mofetil. The healthy donor cohort (N = 9) was 63% female.

Myelomonocytic THP-1 cells and embryonic kidney HEK293T cell lines were maintained in culture medium containing supplements and 10% heat inactivated FBS (complete medium). HEK293T cells (lacking CD33/LAIR-1) were transfected using Lipofectamine 2000 (Invitrogen) for 24 hrs with control/empty pcDNA plasmid (Origene) or pcDNA plasmid containing either hu CD33M (NM_001772;Origene), hu CD33m (NM_001082618) or hu LAIR-1 (generously provided by John Coligan, National Institutes of Health, Bethesda, MD). Transfection was verified by immunoblot analysis using anti-Flag Abs (Sigma-Aldrich) and cell surface expression of LAIR-1/CD33M/CD33m on HEK293T cells was confirmed by flow cytometry using Abs detecting LAIR-1, and CD33 (HIM3-4; WM53). Mono-DCs and mono-macrophages were generated from freshly prepared normal blood monocytes with GM-CSF + IL-4 and M-CSF, respectively as described by us^8, 12^.

### Flow Cytometry

Cells were suspended in buffer containing PBS, BSA, NaN_3;_ then incubated in blocking reagent (1 mg/ml hu gamma block, Sigma) for 15 min on ice. For direct assays, cells were then incubated with experimental or isotype matched control Abs for 35 min on ice. For indirect assays, cells were incubated with primary Abs for 35 min then secondary Abs for 25 min on ice prior to fixation in 1% formalin. Events were acquired on BD LSR Fortessa™ or FACS Calibur (BD Biosciences). For detecting C1q binding to the cell surface, cells were suspended in staining buffer (with cations/without blocking reagent), incubated with biotin-C1q for 40 on ice, and then with streptavidin conjugated-PE-Cy7. Analysis was performed using FlowJo software (Treestar), results are shown as mean fluorescence intensity (MFI) and/or percent positive.

### Slot Blot Assay

A microplate filtration device (Schleicher and Schuell) was used to immobilize either whole C1q, C1q fragments, or CD33 (m/M) on nitrocellulose membranes in protein-protein overlay assays previously described by us^12^. Soluble binding partners, either C1q or CD33 labelled with biotin, were detected using streptavidin conjugated Infrared 800 (LI-COR); hu albumin was the control.

### Proximity Ligation Assay

The Duolink assay (Sigma-Aldrich) was used to study if exogenous C1q promotes molecular association of LAIR-1 and CD33 on the cell surface. Freshly isolated monocytes were either untreated or treated with C1q (20 ug/ml) for 40 min; resuspended in PBS, fixed in paraformaldehyde and then deposited onto slides by cytocentrifugation (Cytospin, Shandon). After staining with primary Abs to CD33 and LAIR-1 of distinct species, species-specific secondary antibodies containing complementary DNA probes were added followed by enzymatic ligation and rolling circle amplification using fluorescently labeled complementary probes. Nuclear counterstaining was performed using DAPI; red fluorescent spots representing protein-protein interaction complexes were detected and visualized using an Axio Image. Z1 ApoTome enabled microscope (Zeiss).

### Phosphorylation Assays

In immunoprecipitation assays studying phosphorylation of CD33 ITIM, monocytes (5 × 10^6^ cells/reaction) were isolated by positive selection (StemCell Technologies), resuspended in ice cold PBS, and then either left untreated or treated with C1q, gC1q, or CLR (25 ug/ml) for 5 min or 0.1 mM pervanadate for 15 min at 37 °C. Lysates were then prepared by incubating for 1 h on ice in 1X RIPA buffer (Pierce, Thermo scientific) containing protease and phosphatase inhibitors (Pierce, Thermo scientific). CD33 was immunoprecipitated by incubating lysates with anti-CD33 Abs (WM53, 4ug/reaction) overnight at 4 °C and then with Dynabeads Protein G (Life Technologies). Proteins were resolved by SDS-PAGE using 4–12% NuPAGE gels (Invitrogen), transferred to nitrocellulose membranes, and immunoblotted with rabbit anti-phosphotyrosine Ab (Upstate bioscience Inc.) or anti-CD33 Ab. Bands were detected using the Odyssey Infrared Imaging system (LI-COR) to detect secondary Abs conjugated with Infrared 680 or 800. In the phosphoarray, isolated monocytes (4 × 10^6^) were placed in serum-free media and treated as above. For simultaneously detecting the relative phosphorylation levels of immunoreceptors, cell lysates were incubated on human phospho-immunoreceptor array membranes as detailed by the supplier (Proteome Profiler # ARY004B; R&D Systems). Phosphorylated (p) proteins were detected using horseradish peroxidase conjugated pan anti-phosphotyrosine Abs. Array signals were visualized using X-ray film, quantified by densitometry analysis, and expressed as average pixel density of duplicate samples.

Assays were performed using pyrogen poor proteins, reagents, chemicals. Culture conditions and reagents were ﻿either tested by us using the limulus amebocyte lysate assay kit (Endosafe, Charles River) or by the manufacturers.

### Statistical Analysis

Results are expressed as the mean +/− standard error (SEM). Two-tailed Student’s t tests as well as one-way analysis of variance (ANOVA) followed by Tukey’s correction for multiple comparisons were used for statistics using Prism (Graphpad software). Significance was defined as P ≤ 0.05.

## Electronic supplementary material

Supplementary material

## References

[1] Nayak A, Pednekar L, Reid KB, Kishore U (2012). Complement and non-complement activating functions of C1q: a prototypical innate immune molecule. Innate immunity.

[2] Fraser, D. A., Laust, A. K., Nelson, E. L. & Tenner, A. J. C1q differentially modulates phagocytosis and cytokine responses during ingestion of apoptotic cells by human monocytes, macrophages, and dendritic cells. *J Immunol* **183**, 6175–6185, doi:jimmunol.0902232 (2009). 10.4049/jimmunol.0902232 PMC2843563 19864605

[3] Lood C (2009). C1q inhibits immune complex-induced interferon-alpha production in plasmacytoid dendritic cells: a novel link between C1q deficiency and systemic lupus erythematosus pathogenesis. Arthritis Rheum.

[4] Santer, D. M. *et al.* C1q deficiency leads to the defective suppression of IFN-alpha in response to nucleoprotein containing immune complexes. *J Immunol* **185**, 4738–4749, doi:jimmunol.1001731 (2010). 10.4049/jimmunol.1001731 PMC3065655 20844193

[5] Castellano G (2007). Immune modulation of human dendritic cells by complement. Eur J Immunol.

[6] Ramirez-Ortiz ZG (2013). The scavenger receptor SCARF1 mediates the clearance of apoptotic cells and prevents autoimmunity. Nature immunology.

[7] Benoit, M. E., Clarke, E. V., Morgado, P., Fraser, D. A. & Tenner, A. J. Complement Protein C1q Directs Macrophage Polarization and Limits Inflammasome Activity during the Uptake of Apoptotic Cells. *J Immunol*, doi:jimmunol.1103760 (2012). 10.4049/jimmunol.1103760 PMC3358549 22523386

[8] Hosszu, K. K., Santiago-Schwarz, F., Peerschke, E. I. & Ghebrehiwet, B. Evidence that a C1q/C1qR system regulates monocyte-derived dendritic cell differentiation at the interface of innate and acquired immunity. *Innate Immun* **16**, 115–127, doi:1753425909339815 (2010). 10.1177/1753425909339815 PMC2846191 19710097

[9] Clarke EV, Weist BM, Walsh CM, Tenner AJ (2015). Complement protein C1q bound to apoptotic cells suppresses human macrophage and dendritic cell-mediated Th17 and Th1 T cell subset proliferation. J Leukoc Biol.

[10] Kouser L (2015). Emerging and Novel Functions of Complement Protein C1q. Frontiers in immunology.

[11] Waggoner, S. N., Cruise, M. W., Kassel, R. & Hahn, Y. S. gC1q receptor ligation selectively down-regulates human IL-12 production through activation of the phosphoinositide 3-kinase pathway. *J Immunol* **175**, 4706–4714, doi:175/7/4706 (2005). 10.4049/jimmunol.175.7.4706 16177118

[12] Son M, Santiago-Schwarz F, Al-Abed Y, Diamond B (2012). C1q limits dendritic cell differentiation and activation by engaging LAIR-1. Proc Natl Acad Sci USA.

[13] Ma W (2012). RAGE binds C1q and enhances C1q-mediated phagocytosis. Cellular immunology.

[14] Crocker PR, Paulson JC, Varki A (2007). Siglecs and their roles in the immune system. Nat Rev Immunol.

[15] Perez-Oliva AB (2011). Epitope mapping, expression and post-translational modifications of two isoforms of CD33 (CD33M and CD33m) on lymphoid and myeloid human cells. Glycobiology.

[16] Raj T (2014). CD33: increased inclusion of exon 2 implicates the Ig V-set domain in Alzheimer’s disease susceptibility. Hum Mol Genet.

[17] Paul SP, Taylor LS, Stansbury EK, McVicar DW (2000). Myeloid specific human CD33 is an inhibitory receptor with differential ITIM function in recruiting the phosphatases SHP-1 and SHP-2. Blood.

[18] Meyaard L (2008). The inhibitory collagen receptor LAIR-1 (CD305). J Leukoc Biol.

[19] Ferlazzo G, Spaggiari GM, Semino C, Melioli G, Moretta L (2000). Engagement of CD33 surface molecules prevents the generation of dendritic cells from both monocytes and CD34 + myeloid precursors. Eur J Immunol.

[20] Orr SJ (2007). CD33 responses are blocked by SOCS3 through accelerated proteasomal-mediated turnover. Blood.

[21] Vitale C (1999). Engagement of p75/AIRM1 or CD33 inhibits the proliferation of normal or leukemic myeloid cells. Proc Natl Acad Sci USA.

[22] Varki A (2009). Natural ligands for CD33-related Siglecs?. Glycobiology.

[23] Kovacs H (1998). Evidence that C1q binds specifically to CH2-like immunoglobulin gamma motifs present in the autoantigen calreticulin and interferes with complement activation. Biochemistry.

[24] Gaboriaud C, Frachet P, Thielens NM, Arlaud GJ (2011). The human c1q globular domain: structure and recognition of non-immune self ligands. Frontiers in immunology.

[25] Ravetch, J. V. & Lanier, L. L. Immune inhibitory receptors. *Science* **290**, 84–89, doi:8872 (2000). 10.1126/science.290.5489.84 11021804

[26] Taylor VC (1999). The myeloid-specific sialic acid-binding receptor, CD33, associates with the protein-tyrosine phosphatases, SHP-1 and SHP-2. The Journal of biological chemistry.

[27] Leffler J, Bengtsson AA, Blom AM (2014). The complement system in systemic lupus erythematosus: an update. Ann Rheum Dis.

[28] Blanco P, Palucka AK, Gill M, Pascual V, Banchereau J (2001). Induction of dendritic cell differentiation by IFN-alpha in systemic lupus erythematosus. Science.

[29] Ding D, Mehta H, McCune WJ, Kaplan MJ (2006). Aberrant phenotype and function of myeloid dendritic cells in systemic lupus erythematosus. J Immunol.

[30] Steinbach F (2000). Monocytes from systemic lupus erythematous patients are severely altered in phenotype and lineage flexibility. Ann Rheum Dis.

[31] Hernandez-Caselles T (2006). A study of CD33 (SIGLEC-3) antigen expression and function on activated human T and NK cells: two isoforms of CD33 are generated by alternative splicing. J Leukoc Biol.

[32] Crespo HJ, Lau JT, Videira PA (2013). Dendritic cells: a spot on sialic Acid. Frontiers in immunology.

[33] Chrostek L (2014). Sialic acid level reflects the disturbances of glycosylation and acute-phase reaction in rheumatic diseases. Rheumatol Int.

[34] Poggi A, Tomasello E, Ferrero E, Zocchi MR, Moretta L (1998). p40/LAIR-1 regulates the differentiation of peripheral blood precursors to dendritic cells induced by granulocyte-monocyte colony-stimulating factor. Eur J Immunol.

[35] Fraser, D. A., Arora, M., Bohlson, S. S., Lozano, E. & Tenner, A. J. Generation of inhibitory NFkappaB complexes and phosphorylated cAMP response element-binding protein correlates with the anti-inflammatory activity of complement protein C1q in human monocytes. *The Journal of biological chemistry* **282**, 7360–7367, doi:M605741200 (2007). 10.1074/jbc.M605741200 17209050

[36] Mizuochi T, Yonemasu K, Yamashita K, Kobata A (1978). The asparagine-linked sugar chains of subcomponent C1q of the first component of human complement. The Journal of biological chemistry.

[37] Lajaunias F, Dayer JM, Chizzolini C (2005). Constitutive repressor activity of CD33 on human monocytes requires sialic acid recognition and phosphoinositide 3-kinase-mediated intracellular signaling. Eur J Immunol.

[38] Courtney AH, Puffer EB, Pontrello JK, Yang ZQ, Kiessling LL (2009). Sialylated multivalent antigens engage CD22 in trans and inhibit B cell activation. Proc Natl Acad Sci USA.

[39] Dillon SP, D’Souza A, Kurien BT, Scofield RH (2009). Systemic lupus erythematosus and C1q: A quantitative ELISA for determining C1q levels in serum. Biotechnology journal.

[40] Ishida A (2014). Negative regulation of Toll-like receptor-4 signaling through the binding of glycosylphosphatidylinositol-anchored glycoprotein, CD14, with the sialic acid-binding lectin, CD33. The Journal of biological chemistry.

[41] Malik M (2013). CD33 Alzheimer’s risk-altering polymorphism, CD33 expression, and exon 2 splicing. J Neurosci.

[42] Griciuc A (2013). Alzheimer’s disease risk gene CD33 inhibits microglial uptake of amyloid beta. Neuron.

[43] Linnartz, B., Kopatz, J., Tenner, A. J. & Neumann, H. Sialic acid on the neuronal glycocalyx prevents complement C1 binding and complement receptor-3-mediated removal by microglia. *J Neurosci* **32**, 946–952, doi:32/3/946 (2012). 10.1523/JNEUROSCI.3830-11.2012 PMC4037907 22262892

[44] Zhang Y (2014). An RNA-sequencing transcriptome and splicing database of glia, neurons, and vascular cells of the cerebral cortex. The Journal of Neuroscience.

[45] Grevink, M. E., Horst, G., Limburg, P. C., Kallenberg, C. G. & Bijl, M. Levels of complement in sera from inactive SLE patients, although decreased, do not influence *in vitro* uptake of apoptotic cells. *Journal of autoimmunity* **24**, 329–336, doi:S0896-8411(05)00042-9 (2005). 10.1016/j.jaut.2005.03.004 15927794

[46] Orbai AM (2015). Anti-C1q antibodies in systemic lupus erythematosus. Lupus.

[47] Pang Y, Yang XW, Song Y, Yu F, Zhao MH (2014). Anti-C1q autoantibodies from active lupus nephritis patients could inhibit the clearance of apoptotic cells and complement classical pathway activation mediated by C1q *in vitro*. Immunobiology.

[48] Tan Y (2009). Detection of anti-C1q antibodies and anti-C1q globular head domain antibodies in sera from Chinese patients with lupus nephritis. Molecular immunology.

[49] Bonaccorsi I (2010). The immune inhibitory receptor LAIR-1 is highly expressed by plasmacytoid dendritic cells and acts complementary with NKp44 to control IFNalpha production. PLoS One.

[50] Colombo BM (2012). Defective expression and function of the leukocyte associated Ig-like receptor 1 in B lymphocytes from systemic lupus erythematosus patients. PLoS One.

[51] Kanakoudi-Tsakalidou F (2014). Simultaneous changes in serum HMGB1 and IFN-alpha levels and in LAIR-1 expression on plasmatoid dendritic cells of patients with juvenile SLE. New therapeutic options?. Lupus.

[52] Tacnet-Delorme P, Chevallier S, Arlaud GJ (2001). Beta-amyloid fibrils activate the C1 complex of complement under physiological conditions: evidence for a binding site for A beta on the C1q globular regions. J Immunol.

