# Supplementary material for: Evidence for C1q-mediated crosslinking of CD33/LAIR-1 inhibitory immunoreceptors and biological control of CD33/LAIR-1 expression
Source: Sci Rep. 2017 Mar 21;7:270. doi: 10.1038/s41598-017-00290-w (PMC5412647; doi:10.1038/s41598-017-00290-w)
Supplement: Supplementary file 1 — Supplementary material [file 41598_2017_290_MOESM1_ESM.pdf]

Supplementary Material

TITLE: Evidence for C1q-mediated crosslinking of CD33/LAIR-1 inhibitory immunoreceptors and biological control of CD33/LAIR-1 expression.

AUTHORS: Myoungsun Son[1], Betty A. Diamond[1], Bruce T. Volpe[1], Cynthia B. Aranow[1], Meggan C. Mackay[1], Frances Santiago-Schwarz[1,*]

Supplementary Figure S1.

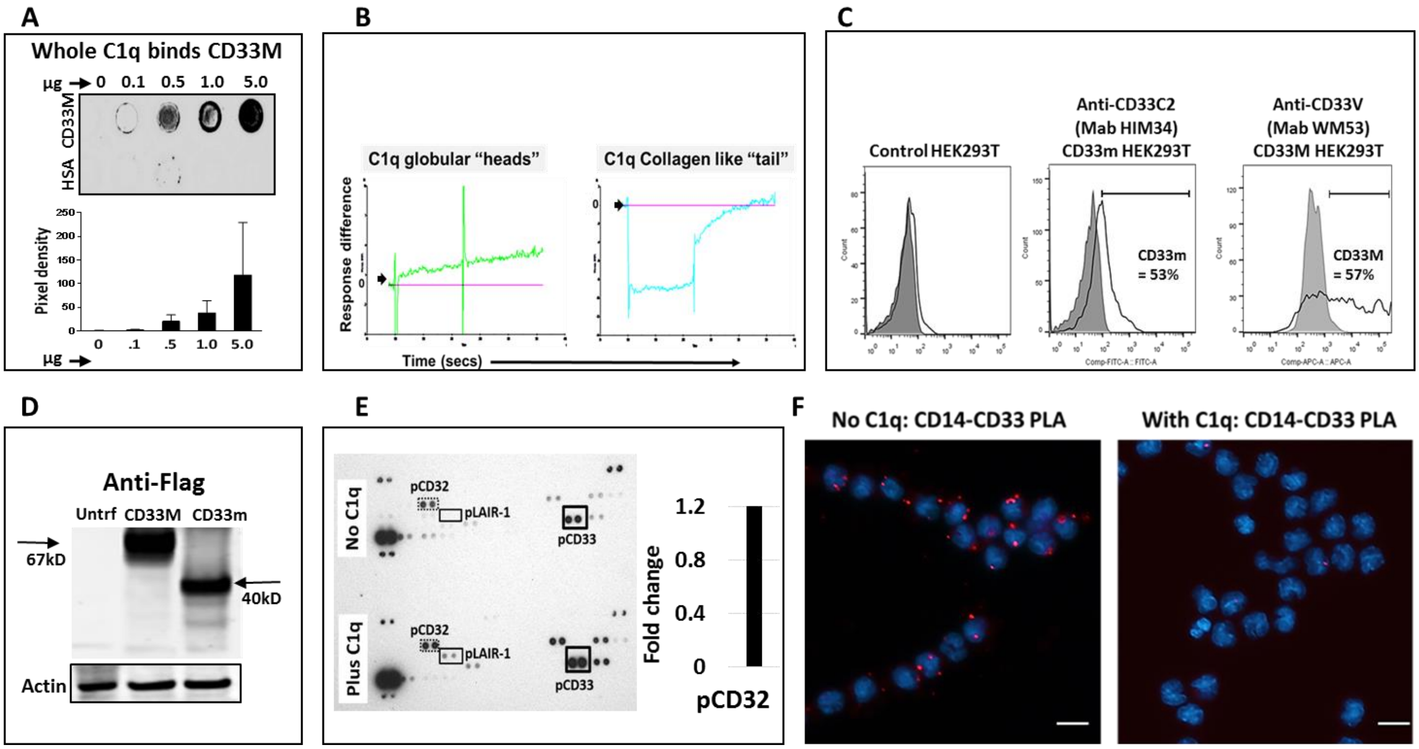

Supplementary Figure S1. (A) Slot blot assay showing that biotin-whole C1q (10 ug/ml) binds to immobilized CD33M in a dose specific manner. Mean ± SE, N=3. (B) surface plasmon resonance (Biacore) sensograms showing increased response difference with gC1q (C1g collagenase digest) but not CLR, substantiating that gC1q and not CLR interacts with CD33. rCD33 (ligand, Novoprotein) was immobilized on a CM5 sensor chip; gC1q&CLR were used as analytes (10 ug/ml). Data were analyzed using BIAeval software. (C) Verification of CD33m, CD33M on the surface of transfected HEK293T cells. CD33m was detected with HIM3-4 mAb specific for CD33C2 epitopes; CD33M was detected with WM53 mAb specific for CD33 V epitopes. Middle and right panels, open graphs represent CD33m, CD33M transfected cells. Left panel, shaded and open graphs represent untransfected (untrf) and mock transfected controls, respectively. Numbers represent percent positive cells. (D) Confirmation of CD33M,CD33m expression in HEK293T cell lysates by immunoblot analysis using anti-Flag Abs (Sigma-Aldrich). (E) Phosphoimmunoreceptor array showing that C1q minimally triggers phosphorylation of CD32 ITAM in normal human (hu) blood monocytes. (F) Proximity ligation assay [PLA, Duolink assay (Sigma-Aldrich.com)] depicting CD14-CD33 cell surface associations on freshly isolated hu monocytes (left panel). Evidence that exogenous C1q (20 ug/ml) displaces CD14-CD33 interactions (right panel). Comparisons were performed in parallel, N=2. Nuclear counterstaining employed DAPI; red fluorescent spots represent CD14-CD33 interaction complexes. Analysis was conducted with an Axio Image.Z1 ApoTome enabled microscope (Zeiss). Bar =10um.

Supplemental Figure 2

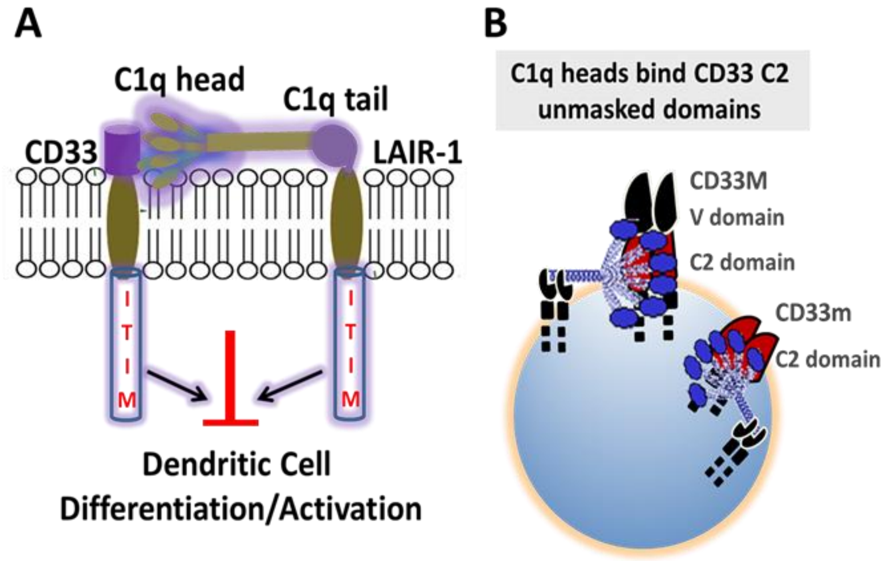

Supplementary Figure S2 (A) Simplified hypothetical scheme showing that C1q cross-links CD33 and LAIR-1 resulting in an inhibitory immunoreceptor signaling complex. The C1q collagen tail (CLR) binds to LAIR-1 while the globular heads (gC1q) bind to CD33 on the monocyte surface to restrict monocyte/DC activation, differentiation/function. Though C1q acting as a molecular bridge between CD33 and LAIR-1 is depicted, given that C1q has repeat binding sites for these proteins on both its globular and collagen regions (and since LAIR-1 and CD33 are abundantly expressed on the monocyte surface) more than one LAIR-1 and CD33 receptor on the cell surface may interact with a single C1q molecule to produce receptor dimerization. Thus, in this setting, C1q also acts as a signal amplification molecule. (B) Representation on the left depicts C1q globular heads binding to unmasked CD33C2-like domains on the CD33M and CD33m isoforms and C1q tails binding to LAIR-1. On the right, sialic acid masking of CD33C2-like domains prevents gC1q binding; V domains, however are still exposed. LAIR-1 expression is not altered by sialylated states, thus allowing C1q's collagen region to engage LAIR-1.

Supplemental Figure 3

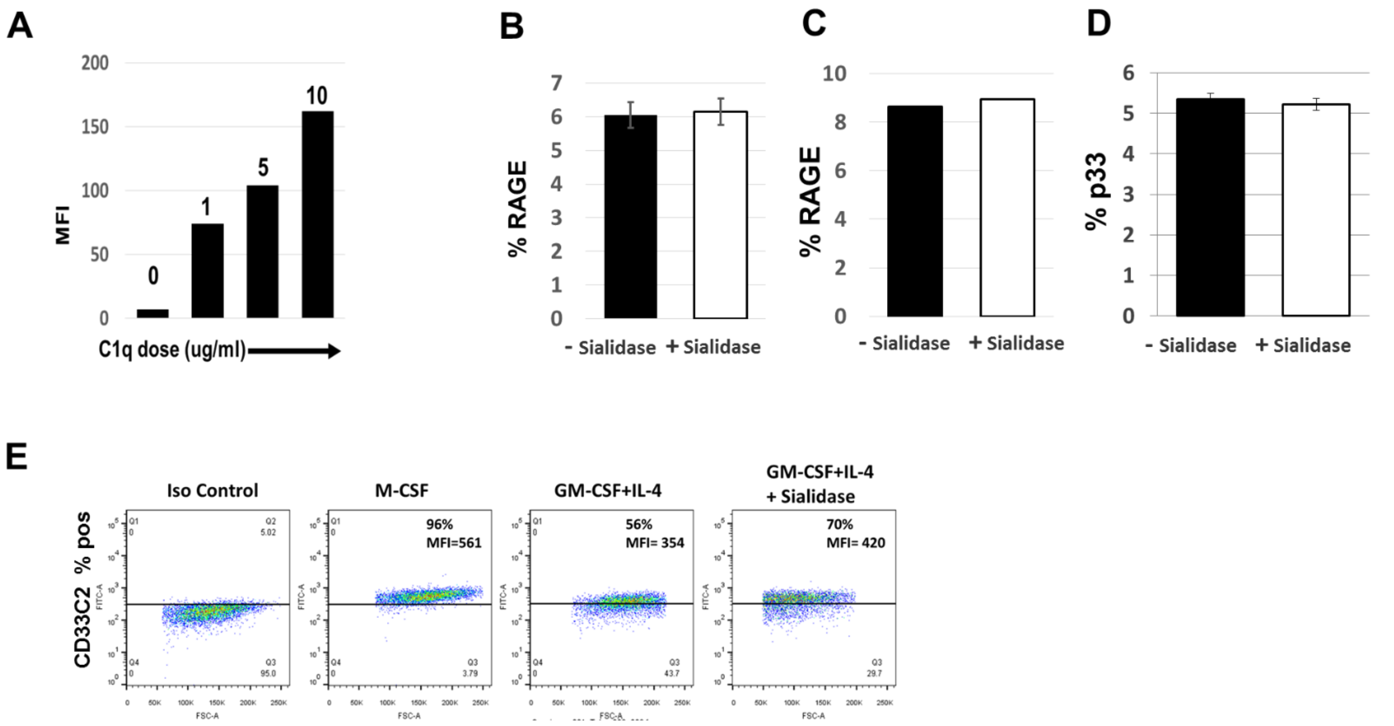

Supplementary Figure S3. A) Dose dependent binding of biotinylated C1q to THP-1 cells. MFI=mean fluorescence intensity of streptavidin-phycoerythrin -Cy7. **(B-D**) C1q binding to THP-1 cells after sialidase treatment is not due to RAGE or p33. B= Percent $RAGE^{pos}$ cells without/with sialidase (mAb MM0520-8D11) N=4-7; P>.05. C= Percent $RAGE^{pos}$ cells without/with sialidase (Rabbit polyclonal, BIOSS) N=1; D= Percent $p33^{pos}$ cells without/with sialidase (mAb 74.5.2) N=2. **E**) Flow cytometry dot plots showing increases in detection of CD33C2 domains (mAb anti-HIM3-4) after sialidase treatment of immature DCs. While M-CSF treatment sustains the level of detectable CD332 domains, GM-CSF/IL-4 does not. For M-CSF, GM-CSF+IL-4, N≥5; GM-CSF+IL-4+sialidase, N=1.

Supplemental Figure 4

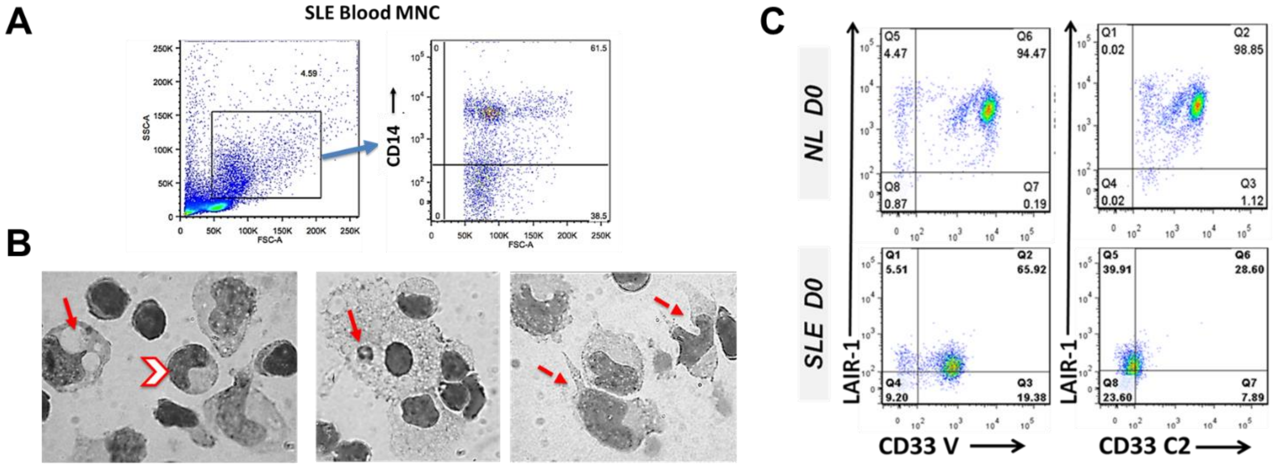

Supplementary Figure S4. A) Gate settings used for SLE patient blood based on forward and side light scatter patterns of mononuclear cells (MNC) on day 0 excluding debris and lymphocytes. CD14 positivity confirmed the presence of monocytes/macrophages in the SLE gates. B) Wright staining of SLE cells displaying a heterogeneous population of activated and differentiated myeloid cell phenotypes. Solid arrows depict highly vacuolated myelomonocytic cells, some containing apoptotic debris; interrupted arrows depict large, irregularly shaped cells; arrowhead denotes a cell with normal blood monocyte features. Original magnification = 60X. C) Typical dual label dot plot analysis showing differences in the expression of CD33C2/CD33V domains versus LAIR-1 on NL vs. SLE monocytes. Gates were set based on isotype controls; a representative experiment (N>3) is shown.
